# Calibration-Free Gaze Interfaces Based on Linear Smooth Pursuit

**DOI:** 10.16910/jemr.13.1.3

**Published:** 2020-03-10

**Authors:** Zeng Zhe, Felix Wilhelm Siebert, Antje Christine Venjakob, Matthias Roetting

**Affiliations:** Chair of Human-Machine Systems, Technische Universität Berlin, Germany; Chair of Work, Engineering & Organizational Psychology, Technische Universität Berlin, Germany; Oculid, Berlin, Germany

**Keywords:** eye movement, eye tracking, gaze, smooth pursuit, gaze interaction, calibration-free

## Abstract

Since smooth pursuit eye movements can be used without calibration in spontaneous gaze interaction, the intuitiveness of the gaze interface design has been a topic of great interest in the human-computer interaction field. However, since most related research focuses on curved smooth-pursuit trajectories, the design issues of linear trajectories are poorly understood. Hence, this study evaluated the user performance of gaze interfaces based on linear smooth pursuit eye movements. We conducted an experiment to investigate how the number of objects (6, 8, 10, 12, or 15) and object moving speed (7.73 ˚/s vs. 12.89 ˚/s) affect the user performance in a gaze-based interface. Results show that the number and speed of the displayed objects influence users’ performance with the interface. The number of objects significantly affected the correct and false detection rates when selecting objects in the display. Participants’ performance was highest on interfaces containing 6 and 8 objects and decreased for interfaces with 10, 12, and 15 objects. Detection rates and orientation error were significantly influenced by the moving speed of displayed objects. Faster moving speed (12.89 ˚/s) resulted in higher detection rates and smaller orientation error compared to slower moving speeds (7.73 ˚/s). Our findings can help to enable a calibration-free accessible interaction with gaze interfaces.

## Introduction

In the past 100 years, eye-tracking technology has evolved significantly. Today, it enables the detection of eye positions and different types of eye movements which is widely used in medical, marketing and psychological research. Apart from the registration of eye movements, person’s gaze has been used to allow users to control interfaces (1). This type of control has a number of advantages over those input methods that rely on physical touch, e.g. hands-free interaction for aseptic environments and increased privacy, since inputs cannot be visually observed by third parties (2). Hence, a large number of applications for gaze-based interaction has been developed for everyday human computer interaction. Gaze typing (3, 4), password input (2, 5), smart watch control (6), map reading (7), and controlling telepresence robots (8) have been proposed as use cases. To trigger an action in gaze-based interfaces, three mechanisms for the selection are prevalent, fixation-based, gesture-based, and smooth-pursuit based selection.

In fixation-based systems, actions, e.g. the selection of objects, are triggered through gaze fixation on an actionable item for a set amount of time (typically called dwell time). Actionable items are fixed on the interface and user’s gaze has to be located on the presented interface. Fixation-based systems suffer from two major disadvantages. First, they require an individual calibration of the eye-tracker, as they depend upon a high accuracy in the registration of the gaze position, to locate user’s gaze in relation to the objects on the interface. With less accuracy, large actionable items with wide separation between objects have to be used, which limits the number of actionable items on the interface. Second, fixation-based systems suffer from the “Midas touch” problem, where actionable items are triggered, although users just look at the actionable item to identify it (9, 10). 

In order to resolve these problems, Drewes and Schmidt (2007) introduced gaze gesture-based interaction (11). In this approach, the completion of a fixed sequence of gaze movements triggers an action. This approach is independent of display space and insensitive to eye-tracking accuracy. However, since users need to learn and remember available gestures, gaze gesture-based interaction is not practical for walk-up interaction, where users have no prior knowledge about the interaction.

## Smooth Pursuit Based Interfaces

In contrast to fixation-based systems, smooth-pursuit based gaze interfaces are composed of moving actionable objects. Instead of an exact location of a user’s eye-gaze on the interface, gaze trajectories are used for object identification, which result from the human eye following a moving object (12). These eye-movements are called smooth-pursuit movements, lending the name to these categories of gaze interfaces. To select an object, users follow a moving object with their eyes, and the resulting gaze trajectory is then compared against the trajectories of moving objects present on the display. Since the detection of trajectories is invariant against its origin location, a calibration phase is not required (13).

To relate the gaze trajectory to moving objects in the interface, Vidal, Bulling, and Gellersen (2013) utilized Pearson’s product-moment correlation in their first implementation of a smooth-pursuit based interface (13). They found that the detection rate was lower when objects in the interface move in linear trajectories compared to circular trajectories. However, detecting horizontal and vertical movement of objects using Pearson’s product-moment correlation can lead to problems. For trajectories that are purely horizontal or purely vertical, there is zero standard deviation when computing the Pearson correlation. The condition that both the highest correlation coefficient of *corr*_*x*_ and *corr*_*y*_ for the objects need to above a threshold is difficult to meet. 

For circular object movements, Esteves et al. (2015) showed that circular gaze trajectories can be well detected in gaze-based smart-watch interfaces (6). For the moving speed of circular object movements, Drewes, Khamis, and Alt (2018) found that speeds between 6 ˚/s and 16 ˚/s result in the highest detection rate (14). Despite their advantages, curved trajectories have been found to be subject to increased gaze deviations compared to the rectangular trajectories consisting of straight lines (15).

Thus, apart from circular object movements, researchers have developed interfaces based on linear pursuit eye movement using other algorithms to improve the detection performance. Cymek et al. (2014) used smooth pursuit eye movements for PIN code input (2). Their interface contained 16 dynamic elements. Each element moved in three segments, combining horizontal and vertical movements. The detection of targets relies on analyzing the combination and classification of gaze trajectory sequences. This interface design was further utilized by Lutz, Venjakob, and Ruff (2015), who developed a gaze typing system based on two-segment pursuit eye movement, called SMOOVS (16). A word prediction functionality integrated by Zeng and Roetting (2018) further improved its typing efficiency (17). In addition, Schenk, Tiefenbacher, Rigoll, and Dorr (2016) developed a system that combined different eye movements, using fixation for object selection, and linear smooth pursuit movements for object activation (18). Freytag, Venjakob, and Ruff (2017) compared two similar smooth-pursuit based interfaces and found that detection accuracy decreased when a large number of objects were presented in the interface (19). So far, there has been no research specifically analyzing the influence of object number and object moving speed in linear trajectory smooth pursuit gaze-based interfaces.

Hence, the main goal of this paper is to develop a deeper understanding for the effect of object number and object moving speeds on the detection performance of gaze interfaces based on linear trajectory smooth pursuit.

## Methods

This study was conducted in the Eye Tracking Laboratory of the Chair of Human-Machine Systems at the Technische Universität Berlin. The goal of the experiment was to understand, how the number of objects and their moving speed influence detection accuracy in linear trajectory smooth-pursuit based interfaces.

### Experimental stimuli

**Figure 1.**
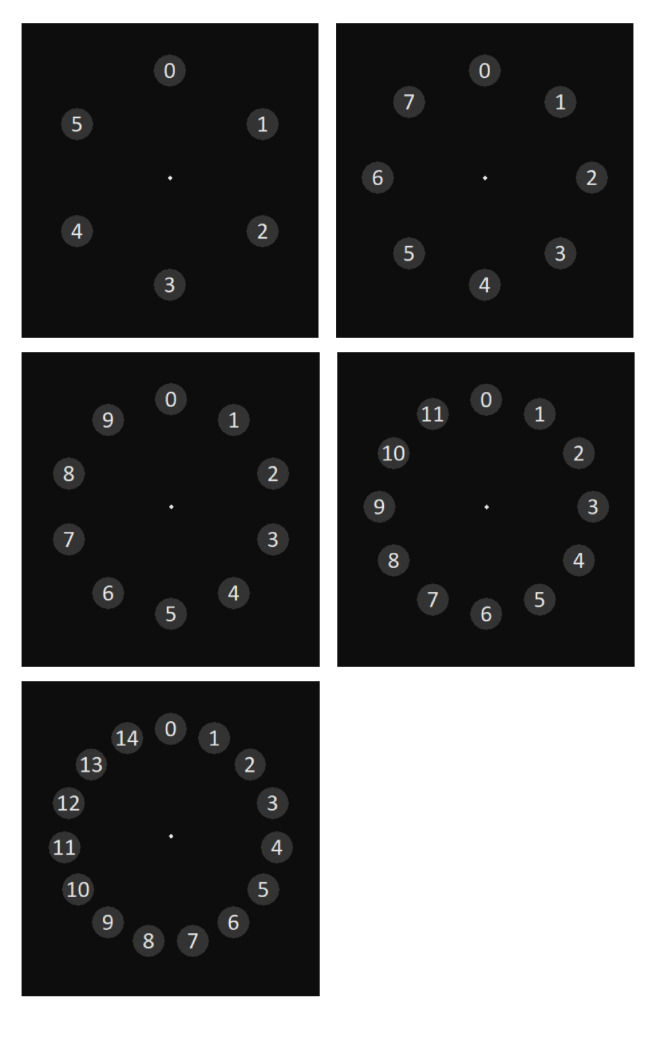
Interface layouts before outward movement of digits. The interface contains 6, 8, 10, 12, and 15 moving objects, respectively.

We developed five interfaces (see Figure 1), which are implemented in Python with the Tkinter GUI package.

The interfaces consist of multiple digits arranged in a circle and vary in the number of digits presented. The ordering of the numbers was constant throughout the experiment (increasing clockwise). The digits move outward in a linear trajectory with constant speed. They are systematically placed, in varied degrees in relation to the center point of the display (see Table 1). 

**Table 1:** Angle size for different interfaces

Number of objects	6	8	10	12	15
Angular division	60˚	45˚	36˚	30˚	24˚
Size of detectable range	55˚	40˚	31˚	25˚	19˚

The diameter of the circles around the digits is 44 pixels (1.13˚ visual angle). The distance from the center of the display to each circle is 150 pixels (3.87˚ visual angle). The interaction with the interface consists of two steps. First, users need to visually perceive the information and search the digit that they want to select. In the second step, users need to follow the chosen digit with their eyes, while all digits move outward.

Since users’ capacity for visual processing is limited, only a certain number of items can be processed at the same time. Research suggests that humans can visually process 20-50 items per second (20). Thus, the digits in the tested interface start to move after 800 ms, ensuring that users have appropriate time to search for a target digit. The gaze points were recorded after the objects start to move. Based on similar research by Vidal et al. (2013) and conventional dwell-time based gaze interactions (21), the duration of the outward movement of digits is set to 500 ms. The recording was ended when the objects stop to move. 

### Classification Algorithm

For evaluation of the gaze trajectory based on smooth pursuit eye movements, we introduce a regression model to detect the linear movements.

This algorithm is based on Orthogonal Distance Regression (ODR), which is a special case of total least squares. It aims to minimize the orthogonal distance from data points to a functional or structural model (22). In this study, we utilized ODR to estimate a linear regression model. The orthogonal distance *r* is defined as the distance from the point to a linear modal. Using ODR, both errors of *X* and *Y* gaze coordinates were taken into account, which can be achieved by estimating the minimization of the sum of the squared distances of r. The function for the calculation of orthogonal distance *r *is: 

**(1):**
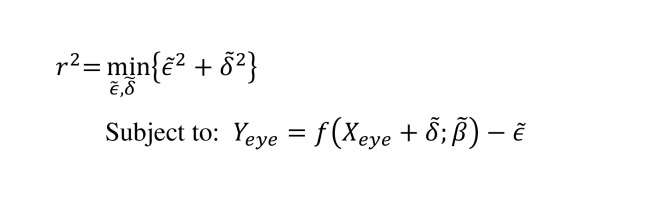


where *X*_*eye*_ and *Y*_*eye*_ are the horizontal and vertical coordinates of gaze data. The random error $\tilde{\delta }$ and $\tilde{\epsilon }$ are corresponding to *X*_*eye*_ and *Y*_*eye*_, respectively. And f refers to a function of *X*_*eye *_ with parameters set $\tilde{\beta }.$

Using ODR, we derived a linear model based on the gaze data. The mean μ and standard deviation σ of the orthogonal distances were calculated. The data points with a distance greater than σ + 3*σ were removed. We iteratively estimate a linear model until no further data points are removed. The angle θ with regard to the linear model is converted from the following function:

**(2):**
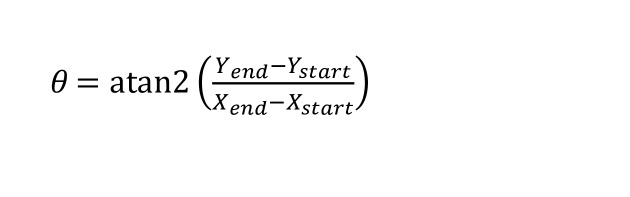


where X_end_ and X_start_ refer to the horizontal coordinates of end and start gaze points. And Y_end_ and Y_start _are the values of the function (1) with X_end_ and X_start_ as input of the function, respectively.

**Figure 2.**
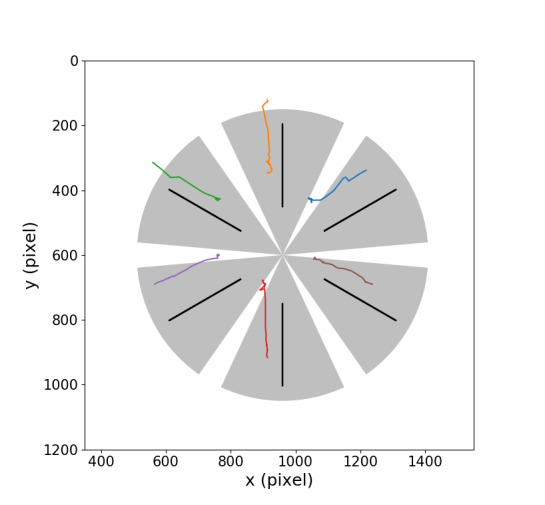
Visualization of the gaze and object trajectories.

An angular criterion was used for target detection. A visualization of the criterion is presented in Figure 2. The black lines show the trajectories of moving digits. Colored lines are trajectory examples of pursuit eye movements from one participant. Gray corridors show the angle ranges that are assigned to individual digits. Gaze trajectories with angles located between two gray angle areas will be recognized as not detected. If the detected gaze angle is within a certain range, it will be detected as the corresponding object. A small angle range (α = 5˚) is defined as a buffer in the middle between two object classification angle areas. If the detected gaze angle is located in this interval, the system will recognize it as not detected. The size of detectable range for different interfaces is shown in Table 1. 

Earlier research has shown that the evocation of smooth pursuit eye movements has a latency range from 80 to 130 ms in relation to the start of object movement (23, 24). There is a relatively long pursuit latency and the eye begins to move later than the moving object. Once the eye starts to move, there are several saccadic movements, allowing the eye to catch up with the moving object (25, 26). In this study, the classification algorithm took this pursuit latency into consideration. Hence, the first 100 ms gaze data of each trial were discarded and only the last 400 ms gaze data were analyzed.

### Experimental Design

This experiment featured a 5×2 within-subjects design. The first factor *number of objects* was varied fivefold (6, 8, 10, 12, or 15 objects in the interface). The factor *moving speed* was varied two-fold, as objects moved either with 7.73 ˚/s (300 px/s) or with 12.89 ˚/s (500 px/s). All experimental conditions were repeated 12 times - that is to say, each participant performed 120 trials in total. 

As dependent variables, two categories of variables were collected, performance measures and measures of subjective experience. For users’ performance, orientation error and detection rates were registered. Orientation error describes the absolute angle difference between the target movement trajectory and the regressed line calculated from eye movement data. The correct detection rate is the ratio between the trials of correct detection and total trials for each participant in each condition. False detection rate refers to the percentage of wrongly detected trials for each participant in each condition, e.g. when the eye trajectory was matched to target “1”, although participants were asked to follow target “2”. For subjective experience, a semi-structured interview was conducted after the experiment. Participants were asked about their preferences for the interfaces regarding the number of objects and object moving speed. They were also asked about possible reasons for their preference.

### Participants

We recruited 25 participants (11 female and 14 male) for this study. Their age ranged from 21 to 46 years old, with a mean of 29.56 years. Seventeen participants had normal vision while eight used vision aids during the study (four wore contact lenses and four wore glasses). Six of the participants had previous experience with gaze interaction. Two participants were left-handed, and 23 participants were right-handed. Participants were rewarded with 5 Euro per visit or alternatively a certification of student experimental hours for attendance.

### Apparatus

A Tobii EyeX screen-based remote eye tracker with a sampling rate of 60 Hz was used to record participants’ eye-movement data. The eye tracker was mounted beneath a 24-inch Dell monitor with a resolution of 1920 × 1200 pixels. All data were collected without any prior calibration phase for individual users. The eye tracker was calibrated by a third person and this setting was used for all the participants. Across all participants, the average gaze estimation error of the eye data was 4˚ visual angle (*SD *= 1.66). The distance between the participants’ eyes to the display was 60 cm (*SD *= 2.67). A chin rest was attached to the edge of the table, corresponding to the horizontal center of the eye tracker and the monitor. The chin rest was used to prevent participants from leaning too close to the display and maintain a constant viewing distance throughout the experiment.

### Procedure

The experiment lasted approximately 30 minutes. After being welcomed, participants were provided with written information about the experiment, and asked to read it carefully. Then, the experimenter explained the eye tracker to participants. Any questions about the experiment were clarified before the participants signed the* Informed Consent Form*. Participants were asked to complete questionnaires including demographic information and experience about eye tracking and gaze interaction. Afterwards, participants were instructed to adjust the chin rest to a comfortable height.

The experiment consisted of one training session and two subsequent test sessions. In each session, a target number was displayed in the center of the screen before the start of individual trials. The moving objects were displayed after the target number was shown 3 seconds. The task for participants was to find the given number in the digits circle and to follow the outward-moving target number with their eyes. In the training session, participants could try out the experimental tasks without data being recorded and familiarize themselves with the interface. Once they fully understood the task, the test sessions were started. In order to balance practice and fatigue effect, the sequence of object moving speed and number of objects was fully randomized. For each experimental condition, i.e. for a given number of objects and a given speed, 12 digits had to be selected. The sequence of the 12 digits was randomized between participants to prevent the effects of sequence. For the interface consisting of 15 digits, not all digits presented in the interface had to be selected in the task. To minimize the potential effects of fatigue, participants took a short break between the two test sessions. A semi-structured interview was conducted after the experiment.

We conducted our experiment with the following hypotheses:

H1: The orientation error will increase with an increased number of objects and conversely decrease with a faster moving speed.

H2: The detection rates for objects will be different regarding number of objects and moving speed.

H3: Users prefer the gaze interface with fewer moving objects and faster object moving speed.

## Results

The gaze data collected during the experiment were analyzed offline. We evaluated the orientation error, the correct detection rate and false detection rate with repeated measures ANOVA at a significance level of α=0.05 . The Mauchly’s test of sphericity was non-significant, so that no correction was needed. A set of Bonferroni corrected t-tests was conducted for pairwise multiple comparisons.

### Orientation Error

**Figure 3.**
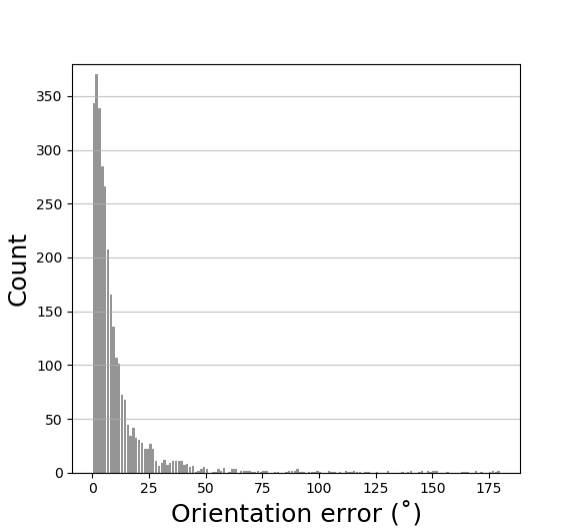
The distribution of raw orientation error throughout the experiment.

Orientation error refers to the absolute angular difference between the target and eye movement trajectory. Figure 3 shows the distribution of orientation errors for all participants and all trials of the experiment. For orientation error, we focus on the performance of smooth pursuit eye movements under a low spatial accuracy. In order to gain further understanding of the orientation error, we excluded outlier data which are three standard deviations away from the mean. We used the remaining 2926 trials for our analysis for orientation error.

**Figure 4.**
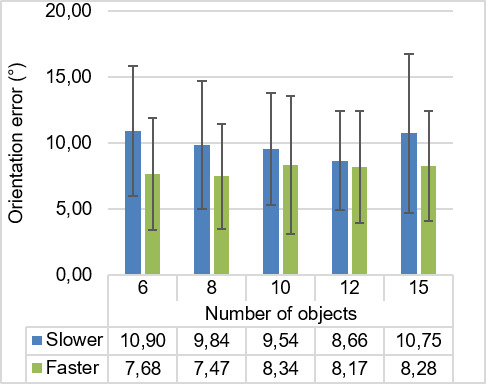
Mean of orientation error in the experimental groups for filtered data.

The grand mean (*M*) of orientation error rate for the filtered data was 7.2˚. Mean values (*M*) and standard deviations (*SD*) for all experimental conditions are visualized in Figure 4. Descriptively, the mean orientation error decreased from 6 objects to 12 objects, while increasing again for 15 objects. Nevertheless, it can be observed that the mean orientation error was higher in experimental conditions with slower moving speed than in conditions with faster moving speed. This descriptive difference can be observed in overall conditions, irrespective of the number of objects presented in the interface.

The ANOVA confirms this effect, the results show that there was no significant main effect of the number of objects on participants’ orientation error ($F\left(4,96\right)=1.27,p=.29,\eta_{p}^{2}=.05$ ). However, participants’ mean orientation error for slower moving speed was significantly greater than faster moving speed $\left(F\left(1,24\right)=30.99,p<0.001,\eta_{p}^{2}=.56\right).$ There was no significant interaction between number of objects and moving speed for correct detection rate (p>.05 ).

**Figure 5.**
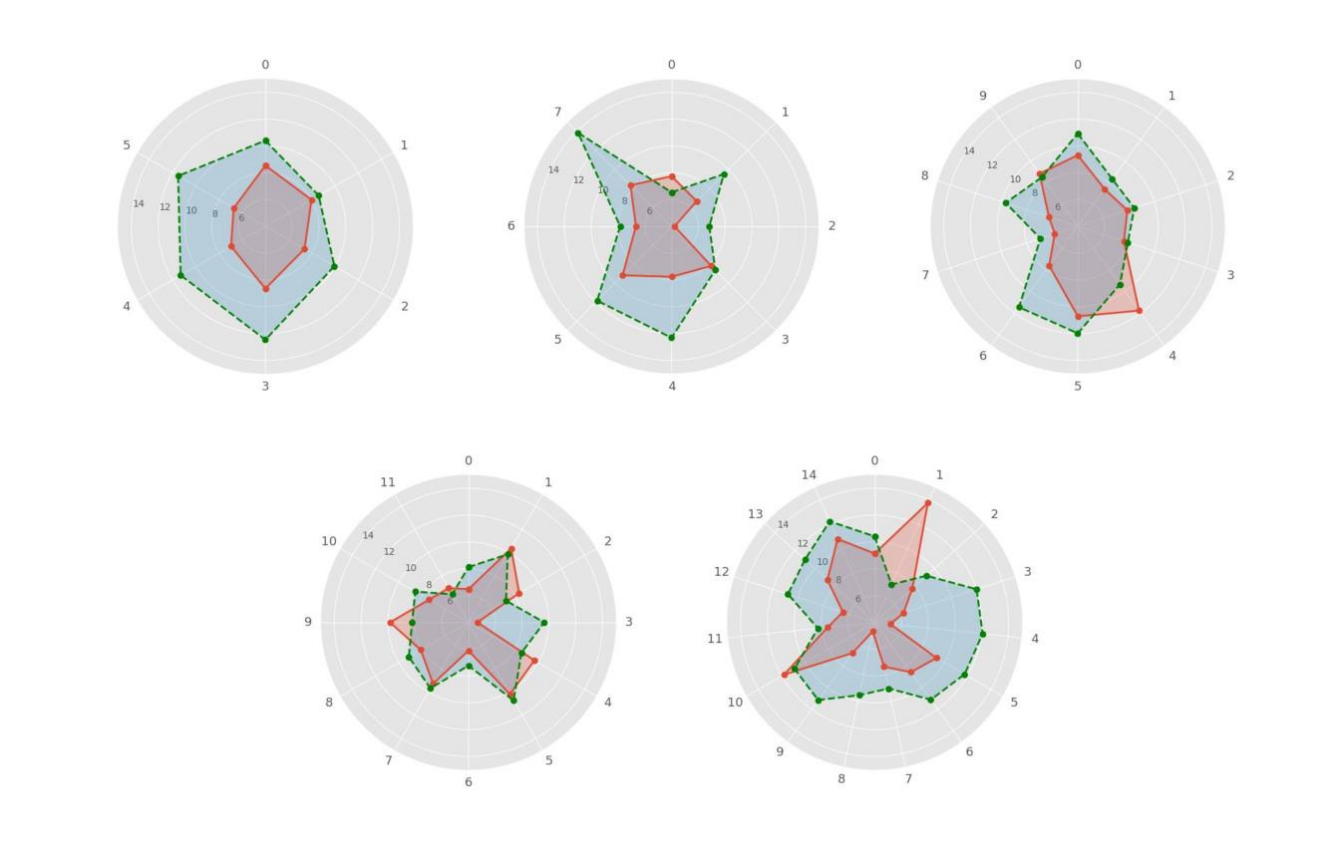
Average object-specific orientation error for all interfaces and the two movement speeds. Green dashed line refers to orientation error of slower moving speed. Red line refers to orientation error of faster moving speed.

The mean orientation error for the different objects in each interface is presented in Figure 5. The orientation error for faster moving speed was generally smaller than the orientation error for slower moving speed. This effect is most pronounced in interfaces with small numbers of moving objects, but becomes less pronounced in interfaces with a large number of moving objects. In addition, the orientation error of some diagonal directions was found larger than cardinal directions for interfaces with 8, 12 and 15 moving objects.

**Figure 6.**
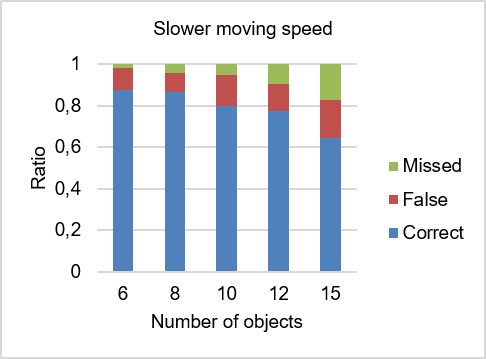
Ratio of detection rates for slower moving speed.

**Figure 7.**
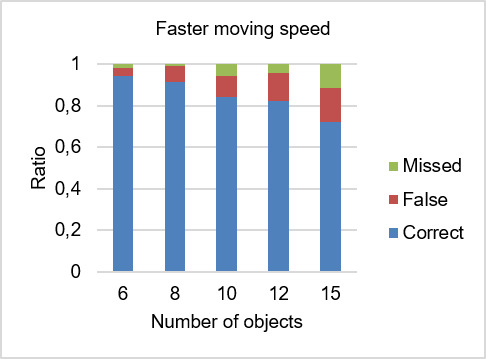
Ratio of detection rates for faster moving speed.

**Figure 8.**
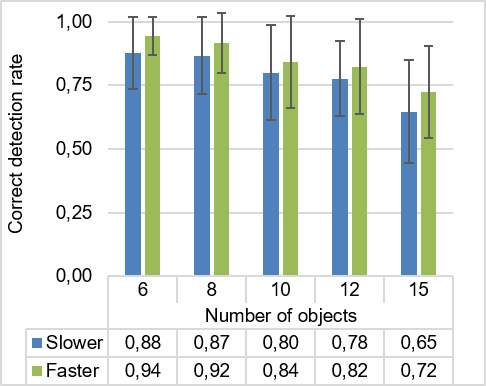
Mean of correct detection rate.

### Detection Rates

The detection rates were analyzed using the raw data set with the consideration of all human errors, i.e. no data was excluded from the analysis. In the following sections, we will evaluate whether the target was detected as being followed (correct detection), detected as another object (false detection) or no object was detected (missed detection). Figures 6 and 7 illustrate the ratio of detection rates.

### Correct Detection Rate

The grand mean (*M*) for the correct detection rate was 0.82. In Figure 8, the mean of the correct detection rate is presented for each condition. The mean of correct detection rates generally decreased with an increasing number of objects. The conditions with faster moving speed had a greater mean for correct detection rate than the conditions with slower moving speed.

The ANOVA shows that there was a significant main effect of the number of objects on participants’ correct detection rate ($F\left(4,96\right)=27.62,p<0.001,\eta_{p}^{2}=.54$ ). Moreover, participants’ mean correct detection rate for faster moving speed was significantly higher than the mean correct detection rate for the slower moving speed $\left(F\left(1,24\right)=13.93,p<0.005,\eta_{p}^{2}=.37\right)$ . There was no significant interaction between number of objects and moving speed for correct detection rate (p>.05 ).

As the results showed a significant effect of the number of objects upon correct detection rate, a set of post hoc *t*-tests was conducted to determine differences between levels. The correct detection rate for 15 objects (M=0.69,SD=0.03) was significantly smaller than those for 6 objects (*M *= 0.91, *SD* = 0.02,p<0.001 ), 8 objects (*M *= 0.89, *SD* = 0.02,p<0.001 ), 10 objects (M=0.82,SD=0.03,p<0.001) , 12 objects (M=0.80,SD=0.03,p<0.005) . The correct detection rate for 6 objects was significantly higher than those from 10 objects(p<0.05) and 12 objects(p<0.001) . Additionally, there were significant differences between 8 and 12 objects(p<0.005) .

### False Detection Rate

**Figure 9.**
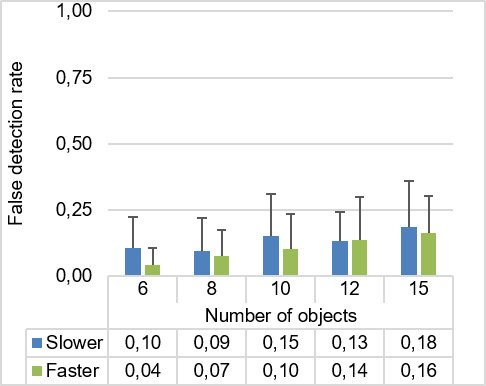
Mean of false detection rate.

A false detection is registered when a gaze trajectory is detected as an object that is not the target object. The false detection rate describes the ratio of all false detections of all presented trials. The grand mean (*M*) for false detection rate was 0.12. The average false detection rates for all experimental conditions are presented in Figure 9. The mean of false detection rates increased with an increasing number of objects. The conditions with slower moving speed had a greater mean for false detection rate than the conditions with faster moving speed.

To gain further understanding of false detected trials, Table 2 compares the number of trials which were falsely detected as adjacent digits (i.e., eye followed “2”, but the gaze trajectory was detected as adjacent digits “1” or “3”.) and the number of all trials which were falsely detected. 

**Table 2:** Count for trials which were detected as adjacent digits and all false detected trials for all experimental conditions.

Number of objects	6	8	10	12	15
Slower (adjacent/all)	23/31	22/28	35/45	29/39	37/55
Faster (adjacent/all)	9/12	19/22	24/30	36/41	37/48

The ANOVA proved that there was a significant main effect of the number of objects on participants’ false detection rate $\left(F\left(4,96\right)=8.85,p<0.001,\eta_{p}^{2}=.27\right)$ . Meanwhile, the object moving speed significantly affects the false detection rate, $(F\left(1,24\right)=6.99,p<0.05,\eta_{p}^{2}=.23$ ). There was no significant interaction between the number of objects and moving speed for false detection rate (p>.05 ).

Pairwise *t*-tests show that the participants had a significantly higher false detection rate with 15 objects (*M *= 0.17, *SD* = 0.03) than with 6 objects (M=0.07,SD=0.02,p<0.005) , and 8 objects (M=0.08,SD=0.02,p<0.005) . Moreover, there were significant differences between 6 and 12 objects (M=0.13,SD=0.02,p<0.005) .

### Subjective Feedback

**Figure 10.**
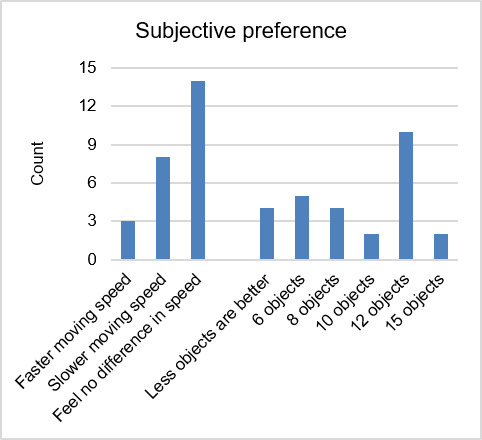
Subjective feedback from participants.

Participants were asked to indicate their preference regarding the number of objects and moving speed. Results of participants’ preference are presented in Figure 10. 

The interface with 12 moving objects was preferred by 10 participants. Open answers to their reasoning behind this preference revealed that participants felt that this interface is similar to the clock face. Five participants chose the interface with 6 moving objects. The 4 participants who indicated a preference for an interface with less than 6 objects reported that they would find it easier to identify the target objects and expected that it would be easier to follow the objects on the interface if there were less than 6 objects on it. In addition, the interfaces with 8 and 15 moving objects were chosen by 2 participants, respectively. 

For the subjective experience of the objects’ moving speed, more than half of the participants did not report a preference, since they did not perceive a difference in moving speed. 8 participants preferred slower moving speed while 3 participants chose faster moving speed as favorite. When asked for reasons behind their preferences, those participants with a preference for slower moving speed reported that following slower moving objects felt easier and required less effort.

## Discussion

This study analyzed how user performance is influenced by the number of objects and object moving speed for gaze interface based linear pursuit movements. 

In our first hypothesis, we anticipated that the orientation error would increase with an increased number of objects and conversely decrease with moving speed. This hypothesis was partly confirmed. We found that the orientation error did not significantly differ between interfaces with varying number of objects. In other words, the pursuit eye movements are not distracted by the increasing number of moving objects and the number of objects has little effect on how well the eye follows the moving target. 

At the same time, the orientation error for interfaces with faster moving speed of objects was significantly smaller than for interfaces with slower moving speed. For slower moving objects, the gaze trajectory does not follow the object path as closely as with faster moving objects. A possible explanation for this difference between moving speed conditions might be that with the same recording time and sample size, the moving distance of faster moving condition is longer than the slower one, thus the ODR regression model for trajectories of faster speed performs better than that of the slower ones.

Additionally, most orientation errors are located in an angle range between 0-30˚. There is only a small number of orientation errors larger than 30˚, which are likely caused by participants’ distraction or a participant’s inability to locate a target object. Since the gaze data were collected using an eye tracker without individual calibration, the orientation errors occurring within this range are mainly due to the accuracy of measuring equipment. 

Although there was no individual calibration for each participant, overall correct detection rates were high. On an individual level, only one participant had a correct detection rate lower than 50%, most likely caused by very thick glasses. For more than two-thirds of false detections, an adjacent digit was detected. In our second hypothesis we expected that the detection rates for objects will be different regarding number of objects and moving speed. We found that the correct detection rate decreased significantly while the false detection rate increased significantly with increasing number of objects in the interface. On the question of differences in object moving speeds, this study found that the correct detection rate increased significantly with the increasing moving speed. The false detection rate significantly lower for faster moving speed. Furthermore, the ratio of trials detected as adjacent digits to all false detection trials is relatively higher for faster than slower moving speed.

The comparison among levels for the number of objects showed that the decrease in correct detection rate was slow between 6 and 8 objects as well as between 10 and 12 objects. But the decrease was larger between 8 and 10 objects as well as between 12 and 15 objects. The correct detection rate of 15 objects was significantly different compared with interfaces with lower number of objects. No significant difference was found between 6 and 8 numbers for both orientation error and detection rates. These differences in detection rates regarding related to the number of objects in the interface may be caused by trials in which participants were not able to find the position of a target object among other objects presented. However, these differences could also be caused by the decrease in the detectable angle range and the limitation of the spatial accuracy. The detectable range was gradually decreasing with the increasing of objects number. For example, the detectable angle range for each object was 55˚ for interface with 6 objects, but the range reduced to 19˚ for interface with 15 objects. Although the target was well followed by the eye, the gaze trajectory could be detected as adjacent objects when the detectable angle was too small and the spatial accuracy was low.

In our third hypothesis we expected that participants would prefer interfaces with fewer objects and fast-moving speed. Our results were inconsistent with the hypothesis. While some participants preferred interfaces with fewer moving objects, the interface with 12 objects was the most preferred interface. The position of the objects played an important role in the subjective evaluation of the interfaces. The similarity of the 12-object interface to a clock face led a number of participants to report a familiarity between the experimental interface and a clock. Concurrently, this might have helped participants to find the target more easily. Future studies should investigate this influence of interface-familiarity on user preference and interaction performance.

While a number of participants did not consciously register the difference in moving speed of objects, some of them preferred slower moving speeds. This is an interesting finding, as it reveals that the subjective experience of users in gaze-based interfaces is not directly linked to a higher performance while using the interface.

## Conclusion and Future Work

In this study, we conducted a controlled laboratory experiment to evaluate the effect of objects number and object moving speed on interaction based on linear smooth pursuit eye movements with no individual calibrated eye tracker. When comparing the number of objects, there was only a little difference in orientation error, but the detection rates decreased with an increasing number of objects. We found the detection of faster moving speed was better than the slower ones. Overall, both the 6 and 8 objects interface with a faster moving speed yielded good user performance. In previous works, six moving directions were frequently used in linear smooth pursuit based interface^ 16,17^. This study shows that the difference between 6 and 8 objects is not significant, both can be well detected by the system. Therefore, it is possible to extend the moving directions of cluster to improve the flexibility of the gaze interface.

### Ethics and Conflict of Interest

The author(s) declare(s) that the contents of the article are in agreement with the ethics described in http://biblio.unibe.ch/portale/elibrary/BOP/jemr/ethics.html and that there is no conflict of interest regarding the publication of this paper. 

### Acknowledgements

The author Zhe Zeng would like to thank the China Scholarship Council (CSC) for financially supporting her PhD study at Technische Universität Berlin, Germany. We acknowledge support by the German Research Foundation and the Open Access Publication Fund of TU Berlin.
